# 

**Published:** 1914-09-26

**Authors:** 


### The Royal Institute of Public Health.

The Council in pursuance of the terms of a Trust, which enables them to award annually a gold medal to a public health medical official at home or abroad, in recognition of conspicuous services rendered to the cause of preventive medicine within the British Empire, have conferred the medal for 1914 upon James Niven, Esq., M.A., M.B., LL.D., medical officer of health for the city of Manchester.

### Gifts and Bequests.

Mr. John Joseph Allen, of Cerne Abbas, Branksome Park, has left £250 to the Bournemouth Royal Victoria Hospital.

Mr. Diedrich Hermann Alten, of Barnet, has left £100 each to King Edward's Hospital Fund for London, the German Hospital, Dalston, N.E., and to the Victoria Cottage Hospital, Barnet.

Mrs. Helen Clunie Sturmy, of Blackheath, has left £500 each to the Blackheath and Charlton Cottage Hospital, the Norwood Cottage Hospital, Dr. Barnardo's Homes, and the London Hospital; and £1,000 to other charities. She also left £50 to her medical attendant, and £50 to her companion.

RATES OF SUBSCRIPTION: Twelve months, 6/6; Six months, 4/-; Three months, 2/-, post free to any address in the United Kingdom. Abroad, Twelve months, 8/8; Six months, 5/-; Three months, 2/6, post free.—Address The Subscription Dept., "THE HOSPITAL," The Hospital Building, 28/29 Southampton Street, Strand, London W.C.

